# Targeted manipulation of grain shape genes effectively improves outcrossing rate and hybrid seed production in rice

**DOI:** 10.1111/pbi.13959

**Published:** 2022-11-26

**Authors:** Xinyu Zhu, Yajun Gou, Yueqin Heng, Wenyan Ding, Yajing Li, Degui Zhou, Xiaoqing Li, Churong Liang, Chuanyin Wu, Haiyang Wang, Rongxin Shen

**Affiliations:** ^1^ State Key Laboratory for Conservation and Utilization of Subtropical Agro‐Bioresources South China Agricultural University Guangzhou 510642 China; ^2^ Guangdong Key Laboratory of New Technology in Rice Breeding, Rice Research Institute Guangdong Academy of Agricultural Sciences Guangzhou 510640 China; ^3^ Institute of Crop Science Chinese Academy of Agricultural Sciences Beijing 100081 China; ^4^ Guangdong Laboratory for Lingnan Modern Agriculture Guangzhou 510642 China

**Keywords:** hybrid rice, stigma exsertion rate, *GS3*, *GW8*, *GS9*

## Abstract

Stigma exsertion rate (SER) of the male sterile line is a key limiting factor for hybrid seed production in rice. Although a large number of quantitative trait loci associated with SER have been reported, few genes have been molecularly cloned and functionally characterized, severely hindering the genetic improvement of SER of the male sterile line and the breeding efficiency of hybrid rice. In this study, we identified three grain shape regulatory genes, *GS3*, *GW8* and *GS9*, as potential candidate genes for targeted manipulation of grain shape and SER. We show that simultaneously knocking out these three genes could effectively increase SER by increasing the ratio of spikelet length/spikelet width and length of stigma and style, without negative impacts on other agronomic traits. Cellular examination and transcriptomic analyses revealed a role of these genes in coordinated regulation of transverse and longitudinal cell division in the pistils. Moreover, we demonstrate that targeted manipulation of these grain shape genes could significantly improve the outcrossing rate in both the ZH11 (a *japonica* variety) and Zhu6S (an *indica* male sterile line) backgrounds. Our results provide new insights into the mechanisms of rice SER regulation and develop an effective strategy to improve SER and out‐crossing rate in rice, thus facilitating hybrid rice production.

## Introduction

Rice is a staple crop for more than half of the world's population (FAO Statistical Databases, 2021). Asian cultivated rice (*Oryza sativa* L.) is domesticated from the wild rice (*Oryza rufipogon* Griff.) and is differentiated into two major ecotypes, *japonica* and *indica* (Huang *et al*., 2012). Since the 1970s, the development of “three‐line” and “two‐line” hybrid systems has contributed significantly to increased rice production and securing global food supply (Peng *et al*., 1999; Yuan and Virmani, 1988; Zhu, 2016). During commercial production of hybrid seeds, plants of the female parent (usually a male sterile line) are planted in rows side‐by‐side with plants of the male parent (normally 5–6 rows of female parent:1 row of male parent), and pollination is aided by humans or mechanicals (such as unmanned‐helicopters). To be commercially profitable, hybrid seed yield should reach no less than 150 kg/mu, and higher yield is desirable for reducing the cost of hybrid seeds for selling to the farmers.

Several characteristics of the female and male parental lines affect the yield of hybrid seed production, including the interval of anthesis between the male and female parents, the stigma exsertion rate (SER) and stigma vigour of the female parental lines, and pollen number/vigour of the male parental lines (Marathi and Jena, 2015; Virmani *et al*., 1982). Among them, SER of the female parental line is believed to be a crucial one. The rice SER is defined as the frequency of exserted stigmas which stay outside after closing of the lemma and palea, and could be gauged by the single stigma exsertion rate (SSE), the dual stigma exsertion rate (DSE) and the total stigma exsertion rate (TSE) (Rahman *et al*., 2017b). As the exserted stigmas could stay viable for 4–6 days to be pollinated, the male sterile lines with high SER would extend the cross‐pollination opportunity by trapping more pollens and overcome non‐synchronous anthesis between the two parental lines, thus elevating the outcrossing ability of the male sterility lines and promoting hybrid rice seed production (Kato and Namai, 1987; Lou *et al*., 2014; Tian, 1993; Wen *et al*., 2009; Yan, 1999; Yuan, 2002). Thus, higher SER (normally above 40–50% TSE) is a preferred trait for the development of a commercially viable male sterile line for hybrid seed production. However, the average SER is <25% in *indica*, <15% in tropical *japonica*, and about 5% in temperate *japonica* (Ling and Xu, 1988; Uga *et al*., 2003; Ying and Zhang, 1989; Zhou *et al*., 2017). Thus, it is often a laborious and time‐consuming process to develop male sterile lines with high SER, which severely limits the breeding efficiency of hybrid rice.

The rice SER is a complex quantitative trait and is easily affected by environmental factors (such as temperature) (Yan *et al*., 2009). Previous studies have shown that SER is largely determined by stigma length (STL), style length (SYL), and the sum of stigma and style length (TSSL) (Dang *et al*., 2020), and thus extensive genetic analyses have been performed to identify SER‐associated loci through phenotyping STL, SYL and/or TSSL. Although more than 40 quantitative trait loci (QTL) for SER have been reported, none of them has been molecularly cloned and functionally characterized, largely due to the difficulty in precise phenotyping and the small additive effects of the individual locus (typically less than 10%) (Bakti and Tanaka, 2019; Li *et al*., 2001, 2014; Liu *et al*., 2015, 2019; Marathi and Jena, 2015; Rahman *et al*., 2017a,b; Tan *et al*., 2020; Uga *et al*., 2003; Zhang *et al*., 2018). Recently, genome‐wide association studies (GWAS) analyses were utilized to identify SER‐associated genes. Notably, three rice grain size regulatory genes, *Grain Size 3* (*GS3*, encodes a G‐protein γ subunit, Mao *et al*., 2010), *Grain Width 2* (*GW2*, encodes a RING‐type E3 ubiquitin ligase, Song *et al*., 2007) and *Grain Width 5* (*GW5*, encodes a novel calmodulin‐binding protein, Liu *et al*., 2017), were identified (Dang *et al*., 2020; Zhou *et al*., 2017). These observations suggest that SER is tightly linked with grain shape. However, the detailed roles of these genes in regulating SER and whether these genes could be utilized to improve SER of the male parent line for hybrid seed production have not been meticulously evaluated.

In this study, we selected three grain shape regulatory genes, *GS3*, *GW8* and *GS9,* as potential candidate genes for targeted manipulation of grain shape and SER. We show that these genes could act synchronously to modulate the spikelet length, spikelet width, stigma length, and style length. Their individual or double knockout plants exhibit improved SER, but the *gs3/gw8/gs9* triple mutant exhibit the most dramatic improvement in SER (TSE reaching ~60%). Moreover, we show that simultaneously knocking out these three genes could effectively improve the outcrossing rate in both *indica* and *japonica* backgrounds. The potential utility of these genes in boosting hybrid seed production, especially in *indica*–*japonica* hybrid seed production, is discussed.

## Results

### Identification of SER regulatory genes by analysing rice grain shape genes

**Figure 1:**
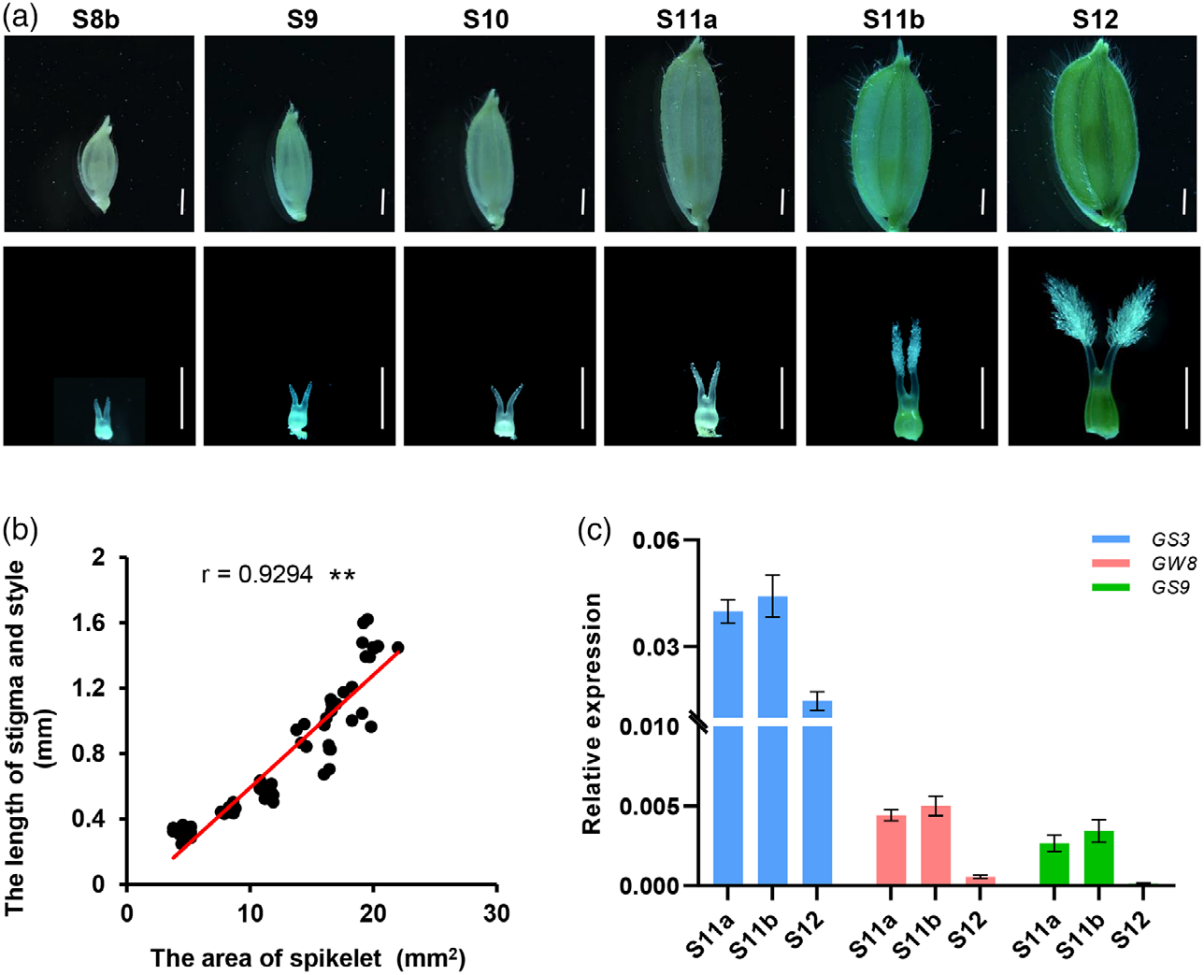
Identification of *GS3*, *GW8* and *GS9* as stigma exsertion regulatory genes in rice. (a) Dynamic change of spikelet and pistil for the ZH11 during spikelet development. S8b‐S12 stages represent different developmental stages based on the reference by Zhang *et al*. (2006) and Zhang *et al*. (2011). Bars = 1 mm. (b) Pearson's correlation between the spikelet area and the total length of stigma and style. *r*, Pearson's correlation coefficient. **P < 0.01. (c) RT‐qPCR analysis of *GS3*, *GW8* and *GS9* in pistils at S11a, S11b and S12 stages. Data are shown as means ± SEM (*n* = 3).

Previous studies have shown that in rice, SER is largely determined by stigma length (STL), style length (SYL), and the sum of stigma and style length (TSSL) (Dang *et al*., 2020) and tightly associated with grain shape (Uga *et al*., 2003, 2010; Zhou *et al*., 2017). Thus, we speculated that these traits might be regulated by a common set of genes. To substantiate this notion, we examined the developmental processes of the spikelet and pistil (containing style and stigma) of ZH11 throughout the S8b ~ S12 stages (Zhang *et al*., 2006, 2011). We found that the spikelet and pistil exhibited synchronous growth, with a sharp increase from S11a to S12 (Figure 1a,b). Pearson's correlation analysis showed that the sum of stigma and style length was significantly and positively correlated with the spikelet area, spikelet length and width (Figure 1b; Figure S1). We then performed a detail analysis of more than 50 rice grain size regulatory genes according to their functions and their genetic effects on grain shape‐related traits (Li *et al*, 2019) (Table S1). Three genes, *GS3*, *GW8* and *GS9*, were selected as they are negative regulators of grain length and/or positive regulators of grain width, and their loss‐of‐function alleles could increase grain quality and confer no adverse effect on other agronomic traits in rice (Fan *et al*., 2006; Wang *et al*., 2012; Zhao *et al*., 2018). Reverse‐ transcription quantitative PCR (RT‐qPCR) assay showed that *GS3*, *GW8* and *GS9* displayed similar expression patterns in the developing pistils, with highest expression at the S11b stage (Figure 1c), coinciding with the maximum growth rate of the spikelet and pistil at this stage. Therefore, we speculated that knocking out these genes may confer increased grain length/width ratio, increased pistil length and consequently increased SER in rice.

### *GS3* , *GW8* and *GS9* synchronously regulate glume shape, pistil growth and the SER in rice

**Figure 2:**
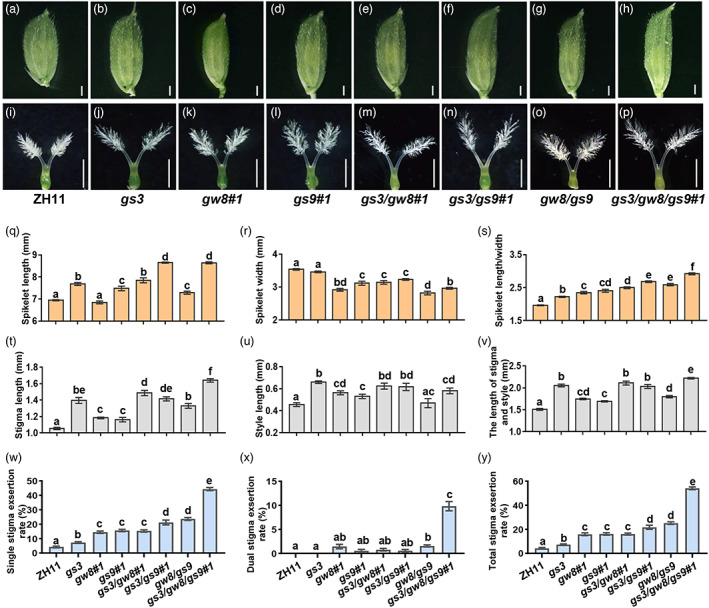
*GS3*, *GW8* and *GS9* synchronously regulate the spikelet, pistil growth and stigma exsertion rate in rice. (a–h) Comparison of spikelet shape among ZH11 and its various knockout combinations of *gs3*, *gw8* and *gs9*. Bar = 1 mm. (i–p) Comparison of pistil shape among ZH11 and its various knockout combinations of *gs3*, *gw8* and *gs9*. Bar = 1 mm. (q–v) Statistical analyses of spikelet length (q), spikelet width (r), spikelet length/spikelet width (s), stigma length (t), style length (u) the length of stigma and style (v), single stigma exsertion (w), dual stigma exsertion (x), total stigma exsertion (y) of ZH11 and its various knockout mutants. Letters above the bars indicate significant differences (*P* < 0.05) as determined by one‐way ANOVA with Tukey's post‐hoc analysis. Data are shown as means ± SEM (*n* = 10).

We thus designed a CRISPR/Cas9 construct aimed to generate various single, double and triple knockout mutants of *GS3*, *GW8* and *GS9* in the *japonica* variety Zhonghua11 (ZH11) background (Figure S2). Multiple single, double and triple mutants harbouring base insertions or deletions were identified through sequencing analysis (Figure S2b–d). Next, we examined the phenotype of spikelet and pistil in various knockout mutants. Compared with the wild‐type (WT) plant ZH11, all the homozygous mutants exhibited increased spikelet length (except for the *gw8* single mutant) but decreased glume width (except for the *gs3* single mutant) (Figure 2a–h,q,r; Figure S3a–f,m,n). Notably, the spikelet length/width ratio was gradually elevated from the single mutant to the triple mutant, suggesting that *GS3*, *GW8* and *GS9* function additively in regulating grain shape in rice (Figure 2s; Figure S3o). In line with the increased spikelet length/width ratio, all the knockout mutants displayed increased stigma length and style length (except for the style length of the *gw8/gs9* double mutant), thus leading to a gradual increase of the total stigma and style length from the single mutant to the triple mutant (Figure 2i–p,t–v; Figure S3g–l,p–r). Consistently, Pearson's correlation analysis showed that the stigma exsertion rate was positively correlated with spikelet length/width ratio and spikelet length, but negatively correlated with spikelet width (Figure S4). As expected, phenotype analysis showed that at anthesis, all the mutated lines exhibited increased SSE, DSE (except for the *gs3* and *gs9* single mutant) and TSE (Figure 2w–y; Figure S3s–u). In comparison to ZH11 with a very low TSE (<5%), the average TSE was increased by 54.92% in *gs3/gw8/gs9#1* and 64.17% in *gs3/gw8/gs9#2* (Figure 2w–y; Figure S3s–u). Noteworthy, the triple mutant produced more slender grains than ZH11with decreased 1000‐grain weight (Figure S5a–d), whereas the number of tillers and grains per plants in triple mutant were significantly increased compared to wild‐type ZH11 (Figure S5k,l). All the improved traits are beneficial for the hybrid seed production. No significant differences were observed for the panicle architecture (including panicle length, number of primary branches and number of secondary branches) and plant height between the triple mutants and wild‐type ZH11 (Figure 5e–j). Together, these findings clearly demonstrate that knocking out these grain shape genes could confer increased SER in rice.

### *GS3* , *GW8* and *GS9* regulate grain and pistil traits by altering cell division

**Figure 3:**
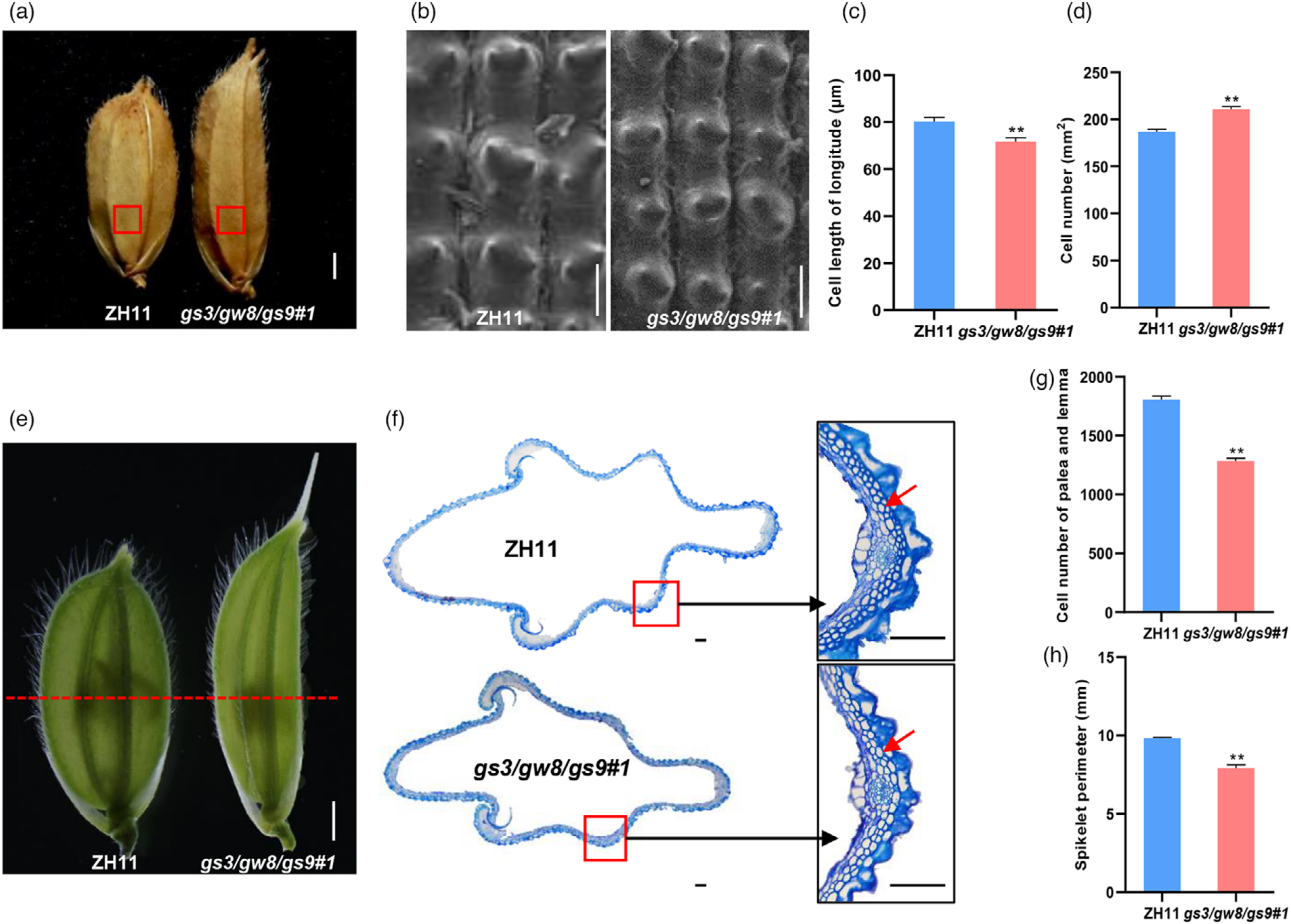
Histological comparison of the spikelet glumes between ZH11 and the *gs3/gw8/gs9#1* triple mutant. (a, b) Scanning electron microscopy analyses of the glume of ZH11 and *gs3/gw8/gs9#1*. (b) is magnified views (enlarged by 200‐fold) of the red boxes areas in (a). (c) The cell length in longitude indicated with red boxes in (a). Bars indicate SEM (*n* = 5). (d) The cell number in the areas indicated with red boxes in (a). Bars indicate SEM (*n* = 5). (e) Spikelet morphology of ZH11 and *gs3/gw8/gs9#1* before anthesis. The yellow dashed line indicates the position of the cross‐sections shown in (f). Bar = 1 cm. (f) Transverse sections of spikelet. The right‐hand images show magnified views of the red boxed region. The red arrow indicates lower epidermal cells. Bar = 100 μm. (g, h) Quantification analyses of cell number (g) and spikelet perimeter (h) of palea and lemma in ZH11 and *gs3/gw8/gs9#1*, Bars indicate SEM (*n* = 3). Asterisks indicate significant differences (**P < 0.01) between ZH11 and *gs3/gw8/gs9#1* by two‐tailed Student's *t*‐test. Data are shown as means ± SEM.

To further examine the cellular changes that occurred in the spikelet and pistil of the *gs3/gw8/gs9* triple mutant, we performed cytological observations in more detail. Scanning electron microscopy (SEM) observations showed that the length of the outer epidermal cells of spikelet glumes in *gs3/gw8/gs9#1* was decreased by ~10% (Figure 3a–c), but an approximate 13% increase in longitudinal cell number compared with the wild‐type ZH11 (Figure 3d), indicating that the increased grain length in *gs3/gw8/gs9#1* is likely resulted from increased cell division in the longitudinal direction. Additionally, transverse sections of palea and lemma showed that *gs3/gw8/gs9#1* contained fewer inner parenchyma cells than the wild‐type ZH11, which may contribute to the decreased transverse spikelet perimeter (by 19%) of the *gs3/gw8/gs9#1* hulls (Figure 3e–h).

**Figure 4:**
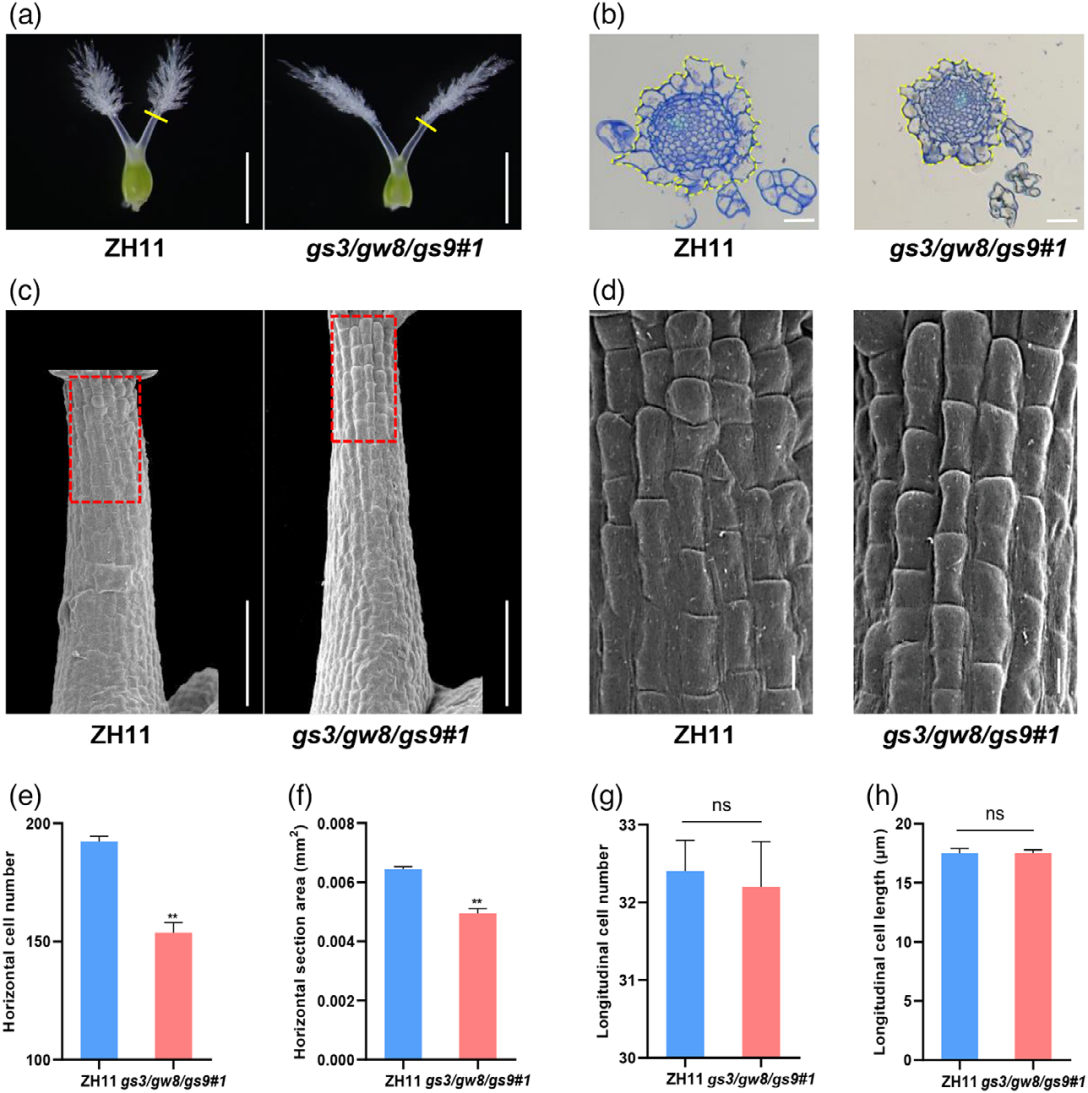
Histological comparison of the styles between ZH11 and *gs3/gw8/gs9#1*. (a) Pistil morphology of ZH11 and *gs3/gw8/gs9#1*. The yellow lines indicate the position of the cross‐sections shown in (b). Bar = 1 mm. (b) Transverse sections of ZH11 and *gs3/gw8/gs9#1* styles. Bar = 20 μm. (c) Scanning electron microscopy analyses of ZH11 and *gs3/gw8/gs9#1* styles. Bar = 100 μm. The dashed boxes highlight the region for comparison of the cell number and cell length in the style between ZH11 and *gs3/gw8/gs9#1*. (d) The magnified views of the boxed regions in (c). Bar = 10 μm. (e, f) The horizontal cell number (e) and area (f) indicated with dashed lines in (b). Bars indicate SEM (*n* = 4). (g, h) Quantification analyses of average parenchymal cell number (g) and cell length (h) shown in (d). Bars in (g) indicate SEM (*n* = 5) and Bars in (h) indicate SEM (*n* = 120). Asterisks indicate significant differences (**P < 0.01) between ZH11 and *gs3/gw8/gs9#1* by two‐tailed Student's *t*‐test. Data are shown as means ± SEM.

Next, we compared the cellular changes within pistil between *gs3/gw8/gs9#1* and wild‐type ZH11. Transverse section analysis of the joint region between stigma and style showed that the number of inner parenchyma cells in *gs3/gw8/gs9#1* was significantly decreased compared with ZH11 (Figure 4a,b,e), thus leading to an approximate 22% decrease in transverse area of the joint region in *gs3/gw8/gs9#1* (Figure 4f). Additionally, transmission electron microscopy (TEM) analysis showed no significant difference in the average cell length and longitudinal cell number of the style between *gs3/gw8/gs9#1* and ZH11 (Figure 4c,d,g,h), implying that the increased style length in *gs3/gw8/gs9#1* is also likely resulted from increased cell division in the longitudinal direction. These results suggest that *GS3*, *GW8* and *GS9* act together to inhibit cell division in the longitudinal direction and promote cell division in the transverse direction of style in rice.

### Transcriptome analysis reveals enrichment of cell proliferation‐related genes in the triple mutant

To further probe the molecular mechanism of action of *GS3*, *GW8* and *GS9* in regulating pistil growth, we performed RNA sequencing (RNA‐seq) to compare the global gene expression profiling between the *gs3/gw8/gs9* triple mutant and ZH11. As the stigma and style of *gs3/gw8/gs9* triple mutant started to display a differential growth rate with that of ZH11 from the S11b to S12 stages (Figure S6), we used the pistils at the S11b stage for transcriptome analysis. Pearson's correlation analysis showed significant correlation between two biological replicates for each sample (Figure S7a). A total of 1498 differentially expressed genes (DEGs) were identified based on the criteria of a 2‐fold change and false discovery rate (FDR) < 0.05 (Dataset S1). Among them, 861 genes were up‐regulated and 637 genes were down‐regulated in the *gs3/gw8/gs9* mutant when compared to ZH11 (Figure S7b). Gene Ontology (GO) term enrichment analysis and KEGG pathway enrichment analysis showed that these DEGs were mainly enriched in multiple processes (Figure S7c,d; Dataset S1, S3). Notably, a large proportion of the DEGs were clustered into cell proliferation–related terms such as ‘cell division’, ‘microtubule binding’, ‘microtubule motor activity’, and ‘cell wall remodeling’ (Figure S7c), consistent with the alteration of cell division detected in the *gs3/gw8/gs9* mutant. We further confirmed the expression profiles of several cell division‐related genes by RT‐qPCR (Figure S7b). Our results suggest that *GS3*, *GW8* and *GS9* cooperatively regulate cell division during pistil development.

### Knocking out of *GS3* , *GW8* and *GS9* significantly increase outcrossing rates of ZH11 and Zhu6S

**Figure 5:**
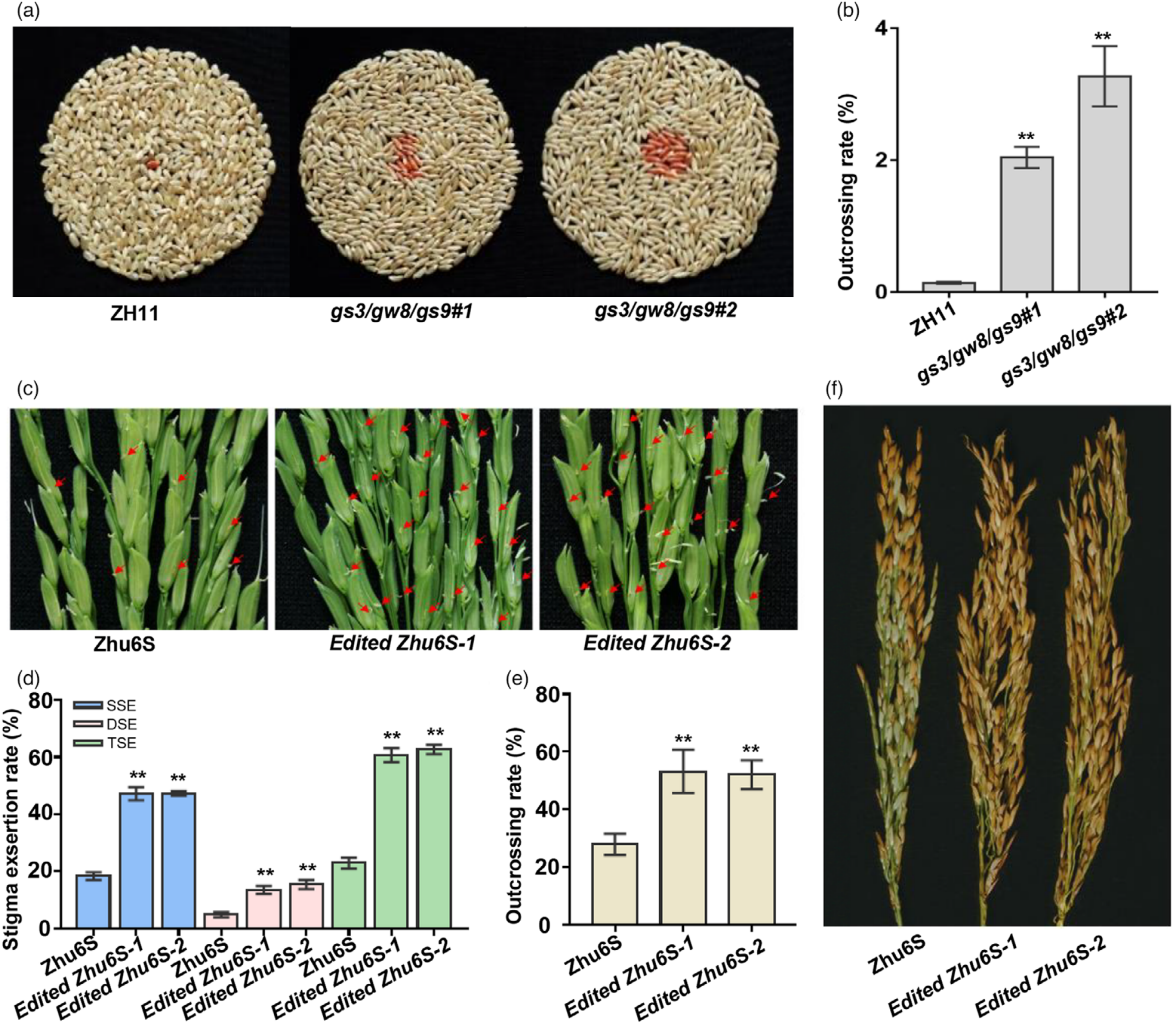
Knocking out *GS3*, *GW8* and *GS9* significantly increase outcrossing rates of ZH11 and Zhu6S. (a) The F_1_ seeds from the crossings of ZH11 or *gs3/gw8/gs9* and the orange‐red‐grained astaxanthin rice. The orange‐red grains represent outcrossing seeds. (b) Measurement of the outcrossing rate of ZH11 and *gs3/gw8/gs9*. Bars indicate SEM (*n* = 4). (c) The stigma exsertion phenotype of Zhu6S and two edited lines (with *GW8* and *GS9* knockout). The red arrows indicate the exserted stigmas. (d) Statistical analysis of stigma exsertion rate shown in (c). Bars indicate SEM (*n* = 10). (e) Measurement of the outcrossing rate of Zhu6S and two edited lines after natural cross‐pollination with Tianfeng B. Bars indicate SEM (*n* = 3). (f) Panicle morphologies of Zhu6S and two edited lines after natural cross‐pollination with Tianfeng B. Asterisks indicate significant differences (**P < 0.01) from ZH11 or Zhu6S, as determined by two‐tailed Student's *t*‐test. Data are shown as means ± SEM.

To test whether the increased SER in the *gs3/gw8/gs9* mutants may promote out‐crossing, we further carried out a field experiment by crossing ZH11 and *gs3/gw8/gs9* with an orange‐red‐grained astaxanthin rice (Zhu *et al*., 2018) as the male parent. The outcrossing rate was evaluated by calculating the percentage of orange‐red grain in the F_1_ seeds (the self‐pollinated seeds are white and the cross‐pollinated hybrid seeds are orange‐red). As expected, both lines of the *gs3/gw8/gs9* triple mutants exhibited significantly higher outcrossing seed setting rates compared with ZH11, when pollinated by the orange‐red‐grained astaxanthin rice (Figure 5a,b). To further evaluate the potential application of loss‐of‐function alleles of *gs3*, *gw8* and *gs9* in hybrid seed production, we selected an elite *indica* male sterile line Zhu6S for genetic improvement. Zhu6S is known to have a low SER (~20%), and thus is not suitable for hybrid seed production. As Zhu6S contained a loss‐of‐function allele of *gs3* (Figure S8a), we simultaneously knocked out *GW8* and *GS9* using the CRISPR/Cas9 technology (Figure S8b). As expected, the edited Zhu6S lines (with *gw8* and *gs9* knockout) exhibited significantly increased SSE, DSE and TSE when compared to the original Zhu6S (Figure 5c,d). The average TSE was increased from 22.87% in the original Zhu6S to 60.65% in the *Edited Zhu6S‐1* and 62.62% in *Edited Zhu6S‐2* (Figure 5d). We further performed a natural outcrossing test by planting the original Zhu6S and its edited lines together with a male fertile rice variety Tianfeng B. As both the original Zhu6S and the edited Zhu6S are male sterile lines, the seeds produced on these plants were hybrid seeds derived from natural cross‐pollination between the sterile lines and Tianfeng B. Notably, the seed setting rate of the edited Zhu6S reached ~50%, which is about two times higher than that of the original Zhu6S (~25%) (Figure 5e,f). These results indicate that manipulation of *GS3*, *GW8* and *GS9* could effectively improve the outcrossing rate of male sterile lines and thus hybrid rice seed production.

## Discussion

The SER trait of the male sterile line is a key limiting factor for hybrid seed production in rice. However, few SER‐related genes have so far been cloned and characterized, severely hindering the genetic improvement of rice SER and the efficacy of hybrid rice breeding. In this study, we demonstrate that besides the previously reported *GS3* (Takano‐Kai *et al*., 2009; Zhou *et al*., 2017), *GW8* and *GS9* are also common regulators of grain shape and SER and that targeted manipulation of these genes could be used to effectively improve SER and out‐crossing rate in rice, thus facilitating hybrid rice production. Our phenotypic analysis showed that there are about 1.7‐fold, 3.7‐fold and 3.8‐fold increases of SER in *gs3*, *gw8*, *gs9* single mutant separately in comparison to ZH11, suggesting that *GW8* and *GS9* play more predominant roles than *GS3* in regulating SER. SER of the double mutants (16.1–25.1%) is obviously higher than that of the single mutants (7.3–16.1%). Notably, SER of the *gs3/gw8/gs9* triple mutant (54.1–64.2%) is significantly higher than that of the *gw8/gs9* double mutant (16.1–25.1%) (Figure 2y; Figure S3u), suggesting that *GS3*, *GW8* and *GS9* may function in a synergistic fashion to regulate SER. Further studies are required to clarify their genetic interaction in SER regulation. In addition, it is worth noting that stigma viability is also an important factor for the success of out‐crossing seed setting (Qi and Wu, 2022). However, whether *GS3*, *GW8* and *GS9* affect stigma vitality also awaits detailed evaluation in future studies. Regardless, our findings hold great potential in targeted improvement of SER for the available male sterile lines as well as facilitating the breeding of new male sterile lines with the superior combining ability and hybrid vigour, thus promoting the utilization and commercialization of hybrid rice.

In addition, as most of the currently planted hybrid rice varieties were intra‐subspecific hybrids of the *indica* subspecies, and their yields have reached a plateau due to the narrow genetic diversity of the parental lines. The *indica*–*japonica* inter‐subspecific hybrid rice has stronger heterosis than intra‐subspecific hybrids of the *indica* subspecies, and has the potential to boost the yield by an additional 20–30% (Peng *et al*., 1999; Yuan and Virmani, 1988). However, utilization of the strong *indica*‐*japonica* inter‐subspecific hybrid vigour is currently restrained by several bottlenecks, including low fertility of the F_1_ plants due to reproduction barriers between the *indica* and *japonica* subspecies, tall plant height/lodging, prolonged life cycle, poor grain quality and low production of hybrid seeds. In particularly, the extremely low level of SER (about 5%) in *japonica* varieties is a major constraint for *indica*–*japonica* inter‐subspecific hybrid seed production. Our findings that both SER and outcrossing rate could be significantly improved in ZH11 (a *japonica* variety) and Zhu6S (an *indica* male sterile line) illustrate an effective approach to overcome this barrier, and thus contributing to the development and commercialization of *indica*–*japonica* inter‐subspecific hybrids in the future.

Besides being an important agronomic trait for hybrid seed production in rice, stigma exsertion is also a key trait of rice domestication. The wild rice ancestor *O. rufipogon* is known to have high SER (80%) and superior outcrossing habit (allogamous), whereas the Asian cultivated rice has greatly reduced SER (on average below 15% in *indica* rice and ~5% in *japonica* rice), rendering the cultivated rice become a strictly selfing (autogamous) species (Xu and Sun, 2021). However, the biological significance and regulatory mechanisms underlying such a transition remain largely mysterious. A recent study demonstrated that *GS3* and *GW5* likely contributed to this change. Based on population genetic analyses, it was inferred that wild rice should have the wild combination of *GW5/GS3* and that in the process of rice domestication, gain of *GS3* function and loss of *GW5* function may have contributed greatly to the change of outcrossing habit of rice to selfing (Zhou *et al*., 2017). In this study, we showed that knocking out *GW8* and *GS9* could significantly increase both SER and outcrossing rate of cultivated rice. Thus, it will be of great interest to explore the genetic diversity of these genes (*GS3*, *GW5*, *GW8* and *GS9*) in wild rice and examine how their haplotype combinations have been artificially selected during rice domestication and modern breeding process. Such analyses may lead to the identification of superior haplotype combinations that confer male sterile lines with desired grain shape/grain quality and high SER for hybrid seed production, with minimal detrimental effects on other agronomic traits, thus contributing to improved breeding efficiency and yield of hybrid rice.

## Methods

### Plant materials and growth conditions

The *GS3*, *GW8* and *GS9* gene were knocked out in the *Oryza sativa* cv. *Japonica* cultivar Zhonghua 11 (ZH11) and the *indica* thermo‐sensitive genic male sterile line Zhu6S through the CRISPR/cas9 technology. The orange‐red‐grained astaxanthin rice was kindly provided by Zhu *et al*. (2018). All rice plants were cultivated in the experimental field at the South China Agricultural University in Guangzhou (23°7′N, 113°15′E) from March to November in 2021 and 2022, and in Lingshui, Hainan (18°22′N, 109°45′E) from November to April in 2021 and 2022.

### Investigation of spikelet shape, pistil‐related traits and agronomic traits

Spikelets of ZH11 and *gs3/gw8/gs9* at different developmental stages were captured and photographed under a light microscope (Zeiss Stemi 508). For investigating the pistil‐related traits, the pistils were carefully separated from the spikelets and photographed under a light microscope (Zeiss Stemi 508). The length and width of the spikelet and the length of stigma and style were measured using the ImageJ software.

To analyse the agronomic traits, the wild type ZH11 and two *gs3/gw8/gs9* lines were grown at the experimental field in Guangzhou from March to July 2021, and eight plants of ZH11 and each mutant line were randomly selected for measurement of grain length, grain width, 1000‐grain weight, panicle length, number of primary branches, number of second branches, plant height, tiller number and grain number.

To calculate the stigma exsertion rate (SER), SER could be gauged by three categories: the single stigma exsertion rate (SSE), the dual stigma exsertion rate and the total stigma exsertion rate (TSE). After anthesis, 10 main panicles from 10 individuals of ZH11, Zhu6S and each mutant line were used to calculate the number of spikelets with single exserted stigma (SES), dual exserted stigma (DES), and no exserted stigma (NES). SER is calculated using the following formulas:

$$\mathrm{SSE}\;\left(\%\right)=\left[\mathrm{SES}/\left(\mathrm{SES}+\mathrm{DES}+\mathrm{NES}\right)\right]\times 100\%.$$

$$\mathrm{DSE}\;\left(\%\right)=\left[\mathrm{DES}/\left(\mathrm{SES}+\mathrm{DES}+\mathrm{NES}\right)\right]\times 100\%.$$

$$\mathrm{TSE}\;\left(\%\right)=\left[\left(\mathrm{DES}+\mathrm{SES}\right)/\left(\mathrm{SES}+\mathrm{DES}+\mathrm{NES}\right)\right]\times 100\%.$$

### Histological analysis

Fresh young spikelet hulls and pistils were firstly fixed in FAA and dehydrated through a graded series of ethanol, then were embedded in epoxy resin (Pon812 Epoxy Embedding Kit; Sigma‐Aldrich, Saint Louis, MO, USA) and polymerized. 3‐μm‐thick sections for pistils and 8‐μm‐thick sections for spikelet hulls were stained with filtered 1% toluidine blue and examined under a light microscope (Nikon, Y‐TV55). The cell number and cell area were measured using ImageJ. To analyse the surface cells of pistils, scanning electron microscopy (SEM) examination was performed as described by Juarez *et al*. (2004) with some modifications. Fresh pistils were fixed in a glutaraldehyde fixative solution (2.5% glutaraldehyde in 0.08 M phosphoric acid buffer) for 24 h at 4°C and then dehydrated through a graded ethanol series (30%, 50%, 70%, 95% and 100%). Dehydrated samples were then dried by a critical point dryer with liquid CO_2_. Finally, the samples were coated with gold palladium using a Desk II sputter coater (Denton Vacuum, Moorestown, NJ) for 45 s before observation under a Hitachi S‐3400N SEM (Hitachi, Kyoto, Japan). To analyse the surface cells of the glumes, mature spikelet glumes were dried and coated with gold palladium, then were observed with a Hitachi S‐3400N SEM with an accelerating voltage of 5 kV.

### Total RNA extraction, RT‐qPCR analyses and RNA‐seq

Total RNA was extracted from the S11b stage pistil using the TRIzol reagent (Thermo Fisher). For RT‐qPCR analysis, approximate 1 μg total RNA of each sample was converted to cDNA using a Hifair® III 1st Strand cDNA Synthesis SuperMix Kit (Yeasen Biotechnology, Shanghai, China) according to the manufacturer's instructions. RT‐qPCR was performed using the Hieff UNICON® qPCR SYBR Green Master Mix (Yeasen Biotechnology) with a LightCycler® 96 System. The *Actin1* gene (*LOC_Os03g50885*) was used as a reference gene. All primer sequences are listed in Table S2.

For RNA‐seq, total RNA of fresh pistils was extracted using Trizol according to the manufacture's protocol. Two biological replicates were used for each sample. A total of 2.0 μg RNA per sample was used to construct cDNA libraries using the mRNA‐seq V3 Library Prep Kit (Vazyme, Nanjing, China) according to the manufacturer's instruction. The Bioanalyzer 2100 (Agilent, Palo Alto, CA, USA) was used to quantify and assess the quality of the RNA samples and cDNA libraries. The paired‐end 2 × 150‐base sequencing was performed on Illumina HiSeq X sequencing platform.

The connectors and low‐quality reads were filtered using Cutadapt (V1.9.1) (Martin, 2011). The processed reads were mapped to the rice Nipponbare reference genome and genes (downloaded from ftp://ftp.ensemblgenomes.org/pub/) using Hisat2 (V2.0.1) (Kim *et al*., 2015). HTSeq (V0.6.1) (Putr *et al*., 2022) was used to count the numbers of reads mapped to each gene. EdgeR Bioconductor package was used to screen differential expression genes (DGEs) at the thresholds of false discovery rate (FDR) ≤ 0.05 and log2 Fold‐Change ≥1. Gene Ontology (GO) and Kyoto Encyclopedia of Genes and Genomes (KEGG) pathway analyses were analysed using an online tool DAVID (https://david.ncifcrf.gov/). Significantly enriched GO terms and KEGG pathway were selected with an empirical *P* value ≤0.05, and the enrichment results were plotted using the ggplot2 package. All DEGs are listed in Dataset S1. Enriched GO terms and KEGG pathway are listed in Dataset S1 and S3, respectively.

### Evaluation of outcrossing rate

To compare the outcrossing rate between ZH11 and the *gs3/gw8/gs9* mutants, ZH11 and two *gs3/gw8/gs9* lines were selected as the female parent, while orange‐red‐grained astaxanthin rice as the male parent. The female and male lines were planted in a 2:4 row ratio, and two rows of female parents were planted in the middle encompassed by four rows of each male line. During the flowering stage, artificial supplementary pollination was performed twice per day. After approximately 30 days, the seeds of the ZH11 and two *gs3/gw8/gs9* mutants were harvested. The outcrossing rate was evaluated by calculating the percentage of orange‐red grain in the F_1_ seeds (including white self‐pollinated seeds and orange‐red hybrid seeds). Four replicates were conducted for each combination.

For the natural outcrossing test, the unedited‐Zhu6S and two edited‐Zhu6S were planted as the female parents together with Tianfeng B (an *indica* variety with a similar heading day as Zhu6S) as the male parent. The female and male lines were planted in a 1:4 row ratio, and one row of female parents was planted in the middle encompassed by four rows of each male line. The crosses between the male and female naturally occurred without being aided by artificial pollination. After approximately 30 days, the seeds of the unedited‐ and edited‐Zhu6S were harvested. The natural outcrossing rate was evaluated by calculating the seed setting rate of the female lines. Six replicates were conducted for each combination.

### Accession numbers

The gene sequence data from this article can be found in the Rice MSU Genome Annotation Release 7 under the following accession numbers: *GW8* (LOC_Os08g41940), *GS3* (LOC_Os04g56400.1), *GS9* (LOC_Os09g27590), *CYCIaZm* (LOC_Os01g59120), *CSLD4* (LOC_Os12g36890), *CYCB2;2* (LOC_Os06g51110), *CDKB2* (LOC_Os08g40170), *CYCA2;1* (LOC_Os12g31810), *PME1* (LOC_Os03g19610), *PME2* (LOC_Os01g20980), *PME31* (LOC_Os11g08750) and *KIN7J* (LOC_Os09g35890).

## Conflict of interest

The authors declare they have no conflict of interest.

## Author contributions

R.S. and H.W. conceived and designed the project. X.Z., and Y.G. performed the research. W.D., Y.L., D.Z., X.L., C.L. and C.W. participated in some experiments. X.Z., Y.G., and Y.H. analysed the data. R.S. and Y.H. wrote the article. H.W. revised the article.

## Supporting information


**Dataset S1** Gene list and FPKM of DEGs in RNA‐Seq.

**Dataset S2** Gene Ontology (GO) term enrichment analyses of differentially expressed genes (DEGs).

**Dataset S3** Kyoto Encyclopedia of Genes and Genomes (KEGG) pathway terms of differentially expressed genes (DEGs).

**Figure S1** Correlation relationship between stigma and style length and spikelet traits.
**Figure S2** *GS3*, *GW8* and *GS9* mutation sites in various knockout lines generated by the CRISPR/Cas9 technology.
**Figure S3** *GS3*, *GW8* and *GS9* synchronously regulate glume, pistil growth and stigma exsertion in rice.
**Figure S4** Correlation relationship between stigma exsertion rate and spikelet traits.
**Figure S5** The effect of *GS3*, *GW8* and *GS9* on several agronomic traits in rice.
**Figure S6** The dynamic change of spikelet and pistil for the ZH11 and *gs3/gw8/gs9#1* during the stages of spikelet development.
**Figure S7** Transcriptome analysis of the pistil of ZH11 and the *gs3/gw8/gs9* mutant at stage 11.
**Figure S8** *GW8* and *GS9* mutation in Zhu6S generated by the CRISPR/Cas9 technology.
**Table S1** List of genes involved in rice grain shape controls.
**Table S2** List of primers used in this study.
