# Supplementary material for: Targeted manipulation of grain shape genes effectively improves outcrossing rate and hybrid seed production in rice
Source: Plant Biotechnol J. 2022 Nov 26;21(2):381–90. doi: 10.1111/pbi.13959 (PMC9884017; doi:10.1111/pbi.13959)
Supplement: Supplementary file 4 — Figure S1 Correlation relationship between stigma and style length and spikelet traits. Figure S2 GS3, GW8 and GS9 mutation sites in various knockout lines generated by the CRISPR/Cas9 technology. Figure S3 GS3, GW8 and GS9 synchronously regulate glume, pistil growth and stigma exsertion in rice. Figure S4 Correlation relationship between stigma exsertion rate and spikelet traits. Figure S5 The effect of GS3, GW8 and GS9 on several agronomic traits in rice. Figure S6 The dynamic change of spikelet and pistil for the ZH11 and gs3/gw8/gs9#1 during the stages of spikelet development. Figure S7 Transcriptome analysis of the pistil of ZH11 and the gs3/gw8/gs9 mutant at stage 11. Figure S8 GW8 and GS9 mutation in Zhu6S generated by the CRISPR/Cas9 technology. Table S1 List of genes involved in rice grain shape controls. Table S2 List of primers used in this study. [file PBI-21-381-s002.docx]

**SUPPORTING INFORMATION**


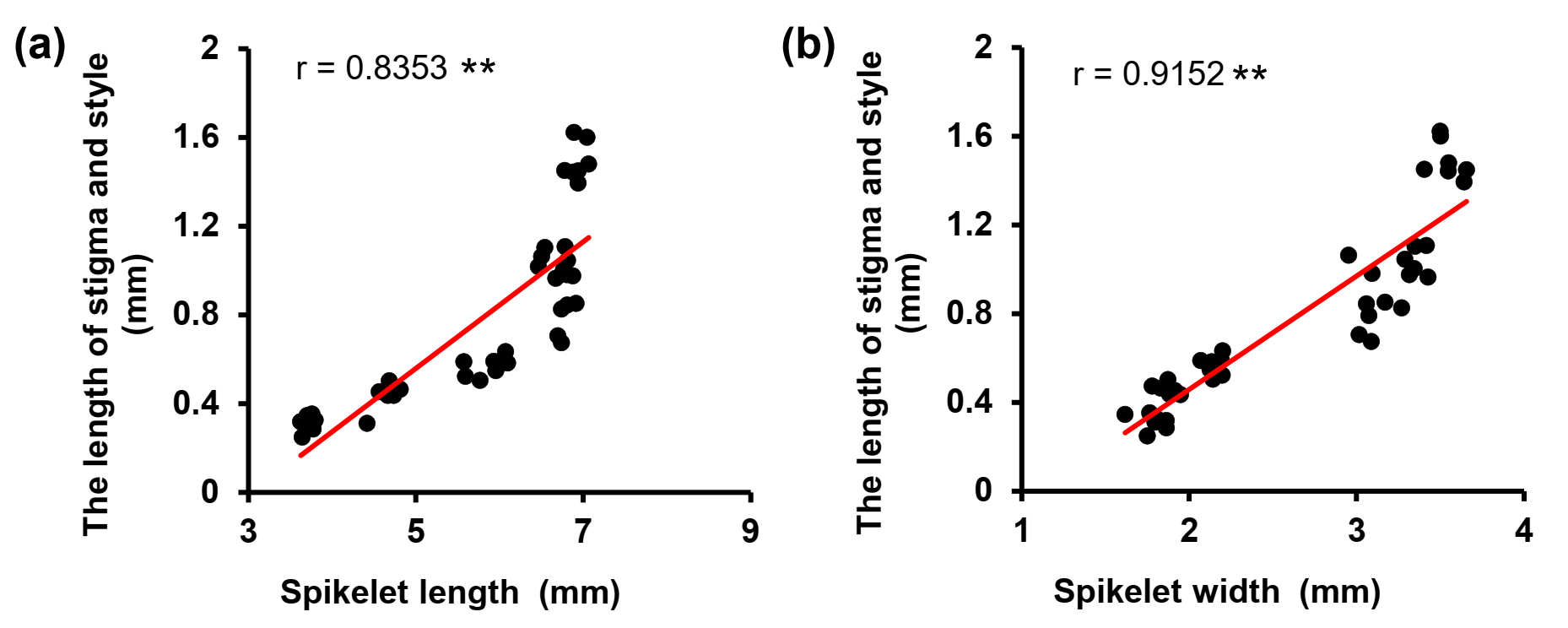


**Supplemental Figure 1.** **Correlation relationship between stigma and style length and spikelet traits.** (a-b) Pearson’s correlation analysis between the spikelet length (a), spikelet width (b) and the total length of stigma and style throughout S8b-S12 stages. r, Pearson’s correlation coefficient. ***p* < 0.01.


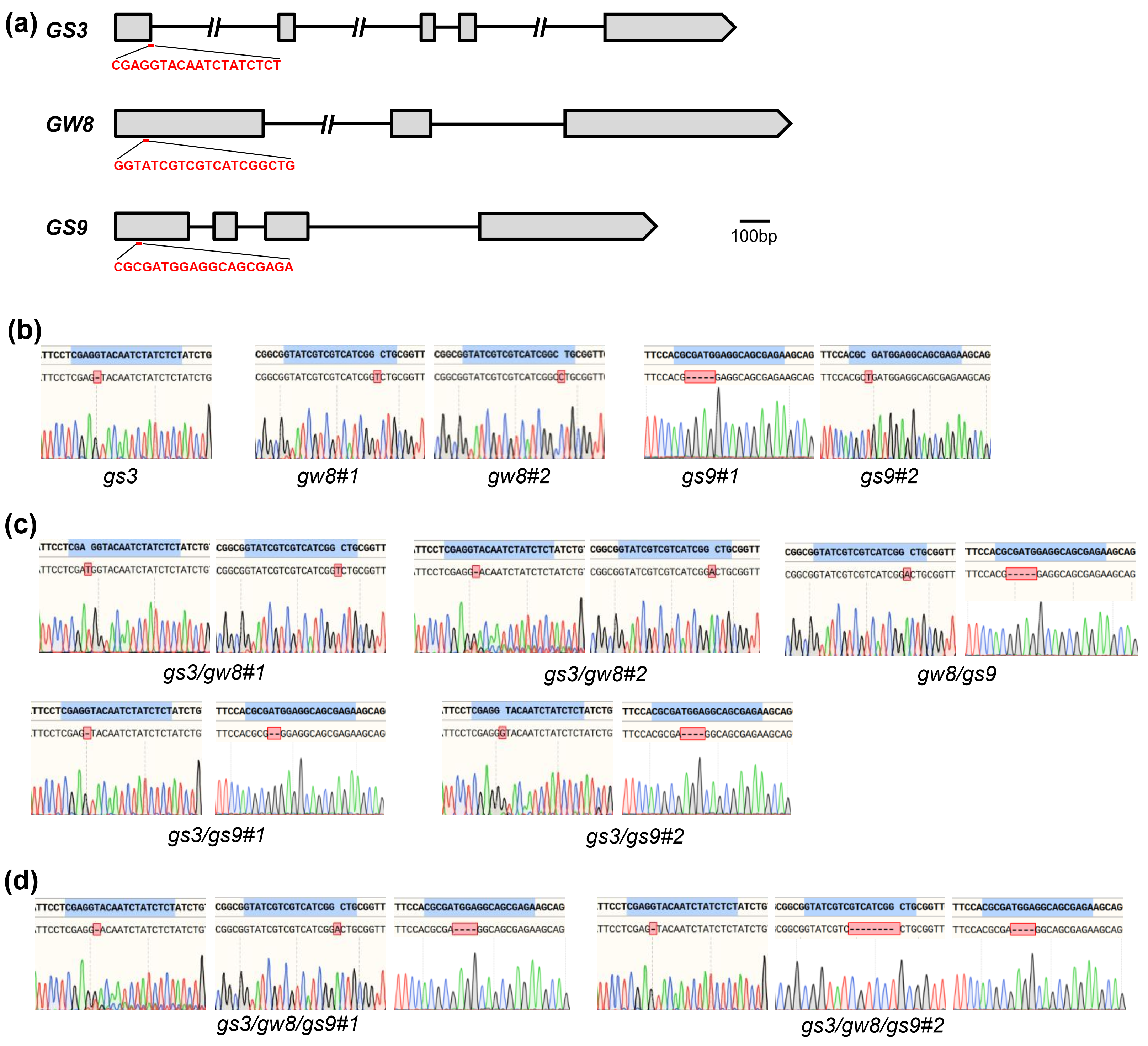


**Supplemental Figure 2. *GS3*, *GW8* and *GS9* mutation sites in various knockout lines generated by the CRISPR/Cas9 technology.** (a) Gene structures of *GS3, GW8* and *GS9* with CRISPR/Cas9 target sites. The red lines indicate the position of the guide RNA. Bar = 100 bp. (b-d) Sequence analysis of various knockout mutants for *GS3, GW8 and GS9.* The target sites are shown with light-blue color, and the mutated information is boxed in pink.


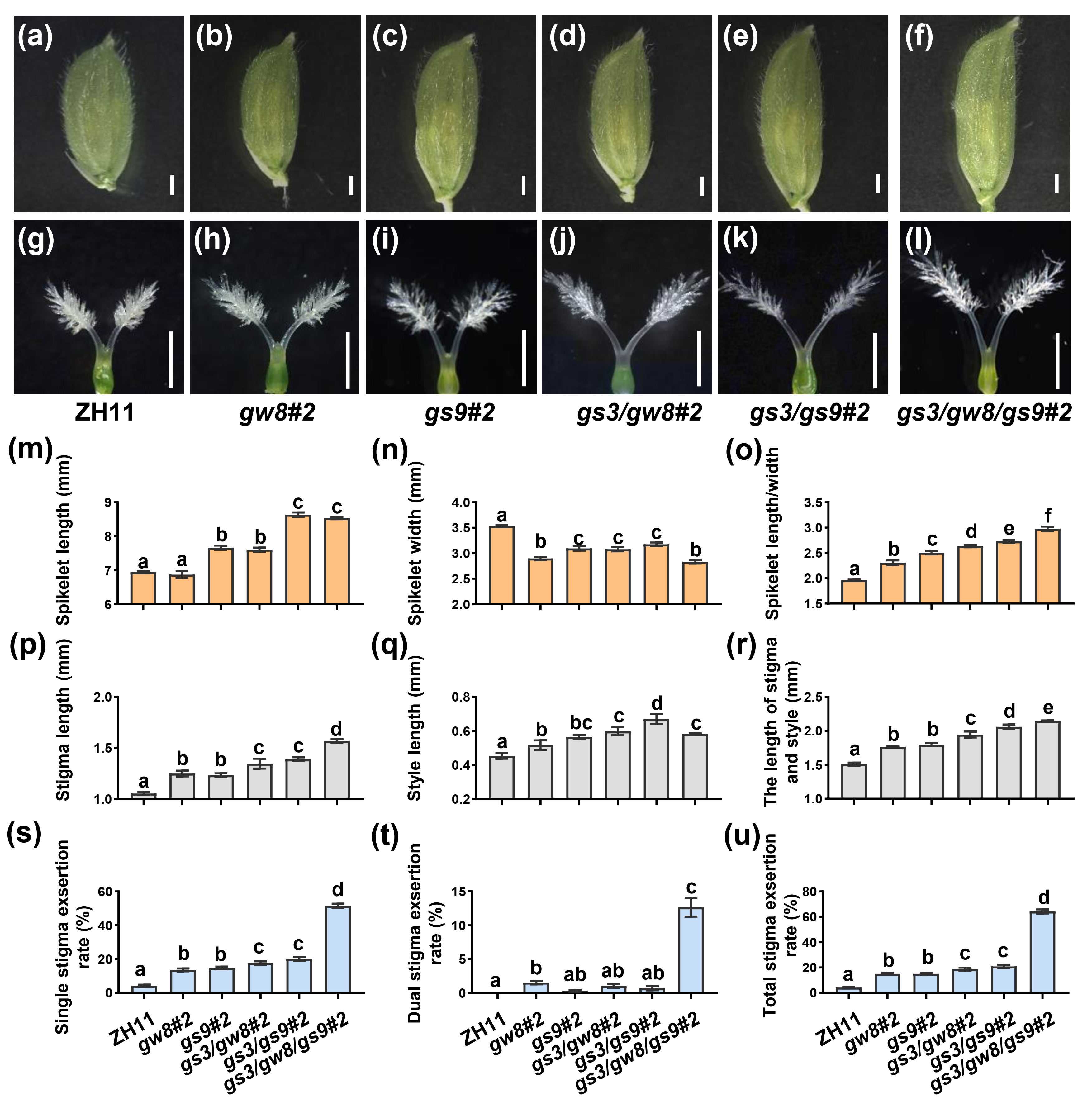


**Supplemental Figure 3. *GS3*, *GW8* and *GS9* synchronously regulate glume, pistil growth and stigma exsertion in rice.** (a-f) Comparison of spikelet shape among ZH11 and its various knockout combinations of *gs3*, *gw8* and *gs9.* Bar = 1 mm. (g-i) Comparison of pistil shape among ZH11 and its various knockout combinations of *gs3*, *gw8* and *gs9*. Bar = 1 mm. (m-u) Quantitative analysis of spikelet length (m), spikelet width (n), spikelet length/ spikelet width (o), stigma length (p), style length (q), the length of stigma and style (r), single stigma exsertion (s), dual stigma exsertion (t), total stigma exsertion (u) of ZH11 and its various knockout mutants. Letters above the bars indicate significant differences (*p* < 0.05) as determined by one-way ANOVA with Tukey’s post-hoc analysis. Data are given as means ± S.E.M (n = 10).


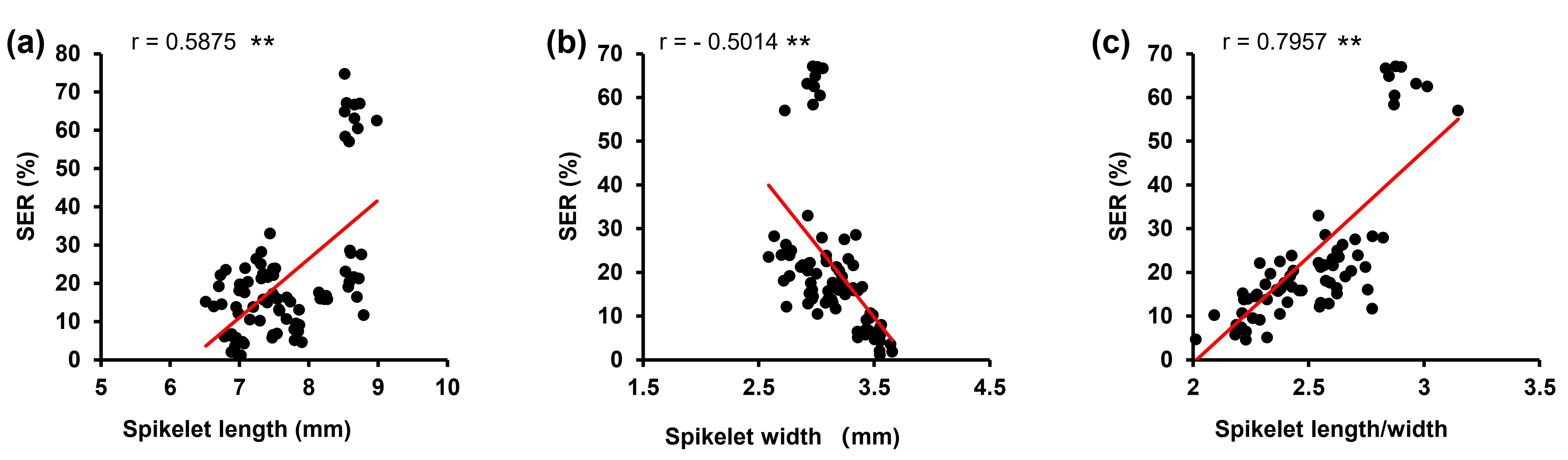


**Supplemental Figure 4.** **Correlation relationship between stigma exsertion rate and spikelet traits.** (a-c) Pearson’s correlation between the spikelet length (a), spikelet width (b), spikelet length/width (c) and the total stigma exsertion rate among ZH11 and its various knockout combinations of *gs3*, *gw8* and *gs9*. r, Pearson’s correlation coefficient. ***p* < 0.01.


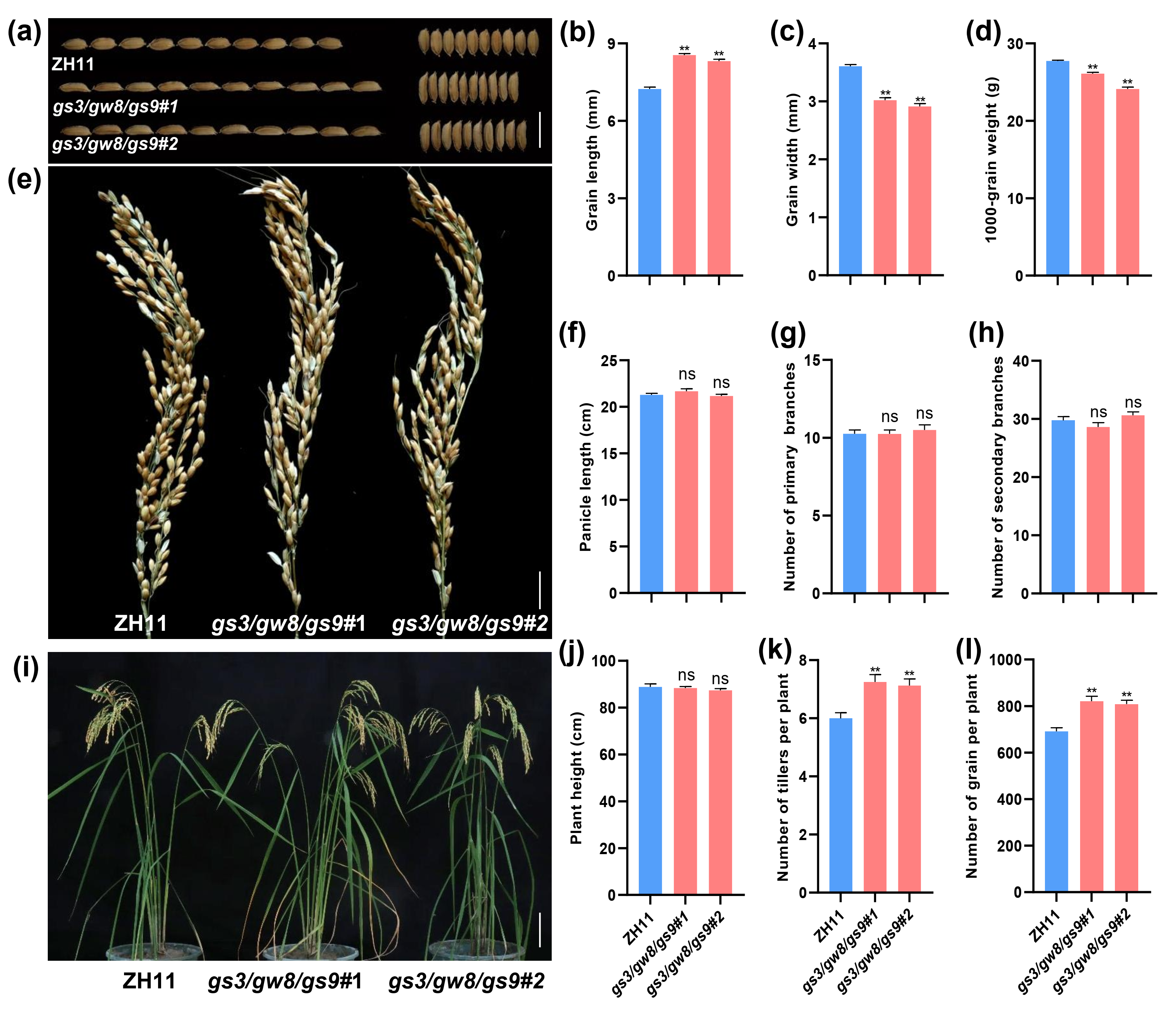


**Supplemental Figure 5. The effect of *GS3*, *GW8* and *GS9* on several agronomic traits in rice.** (a-d) Comparison of grain shape (a), grain length (b), grain width (c) and 1000-grain weight (d) among of ZH11 and the *gs3/gw8/gs9* mutants. (e-h) Comparison of panicle shape (e), panicle length (f), primary branch number (g) and secondary branch number (h) among of ZH11 and the *gs3/gw8/gs9* mutants. (i-l) Comparison of gross morphologies (i), plant height (j), tiller number (k) and grain number per plant (l) among of ZH11 and the *gs3/gw8/gs9* mutants. Bar = 1 cm in (a) and (e), 10 cm in (i). “*” and “**” indicate statistical significance between control plants and transgenic events at *p* < 0.05 and *p* < 0.01 respectively. Data are shown as means ± S.E.M. n = 8 in (b-c), (f-h) and (j-l), n = 5 in (D).


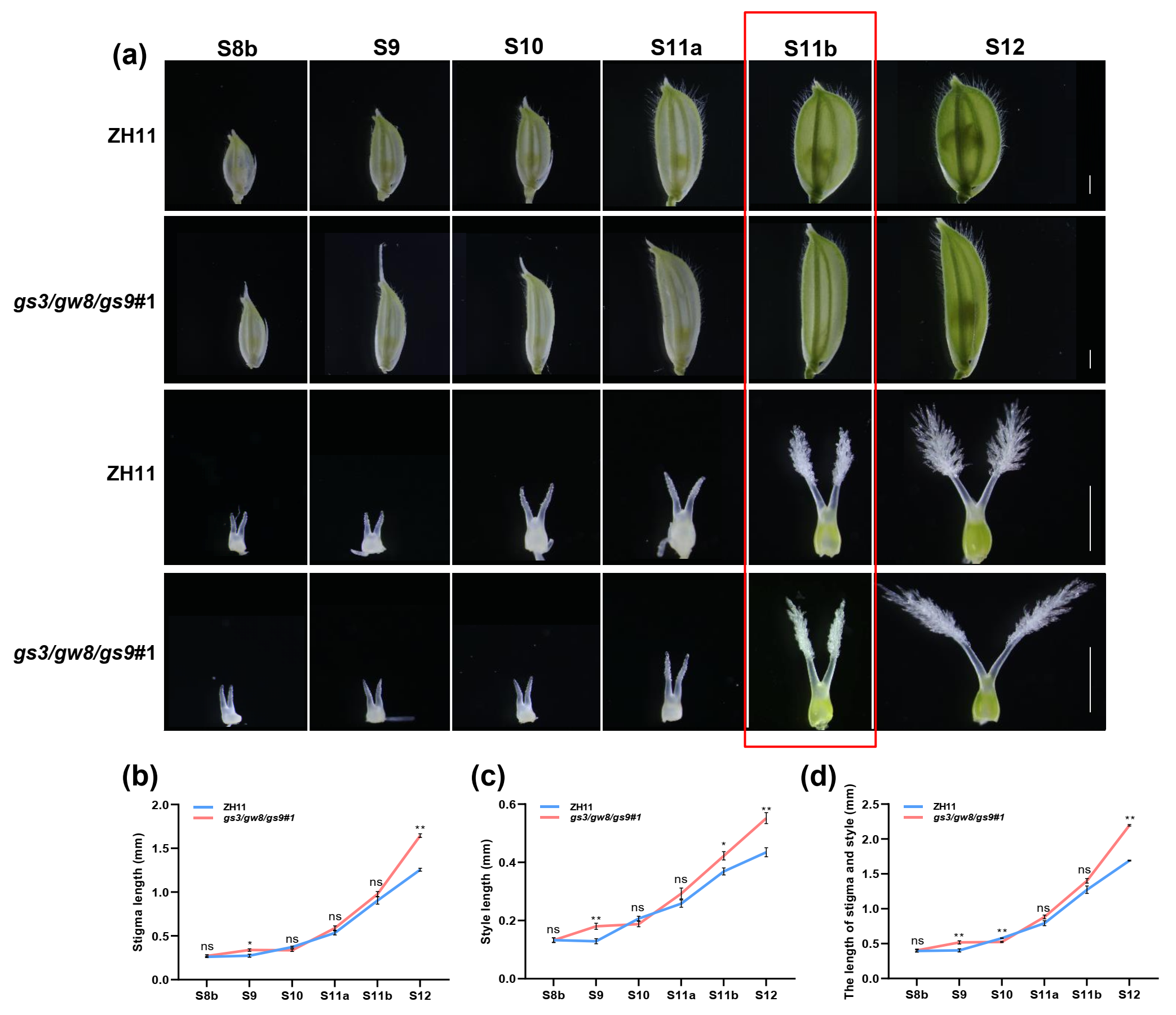


**Supplemental Figure 6. The dynamic change of spikelet and pistil for the ZH11 and *gs3/gw8/gs9#1* during the stages of spikelet development.** (a) Comparison of spikelet shape (the upper two lanes) and pistil shape (the bottom two lanes) between ZH11 and *gs3/gw8/gs9*#1 from S8b-S12 developmental stages (Zhang *et al*., 2006; Zhang *et al*., 2011). Red box indicates spikelet and pistil at S11b stage. Bar = 1 mm. (b-d) Comparison of stigma length (b), style length (c), the length of stigma and style between ZH11 and *gs3/gw8/gs9*#1 from S8b-S12 developmental stages. Data are shown as means ± S.E.M (n = 5).


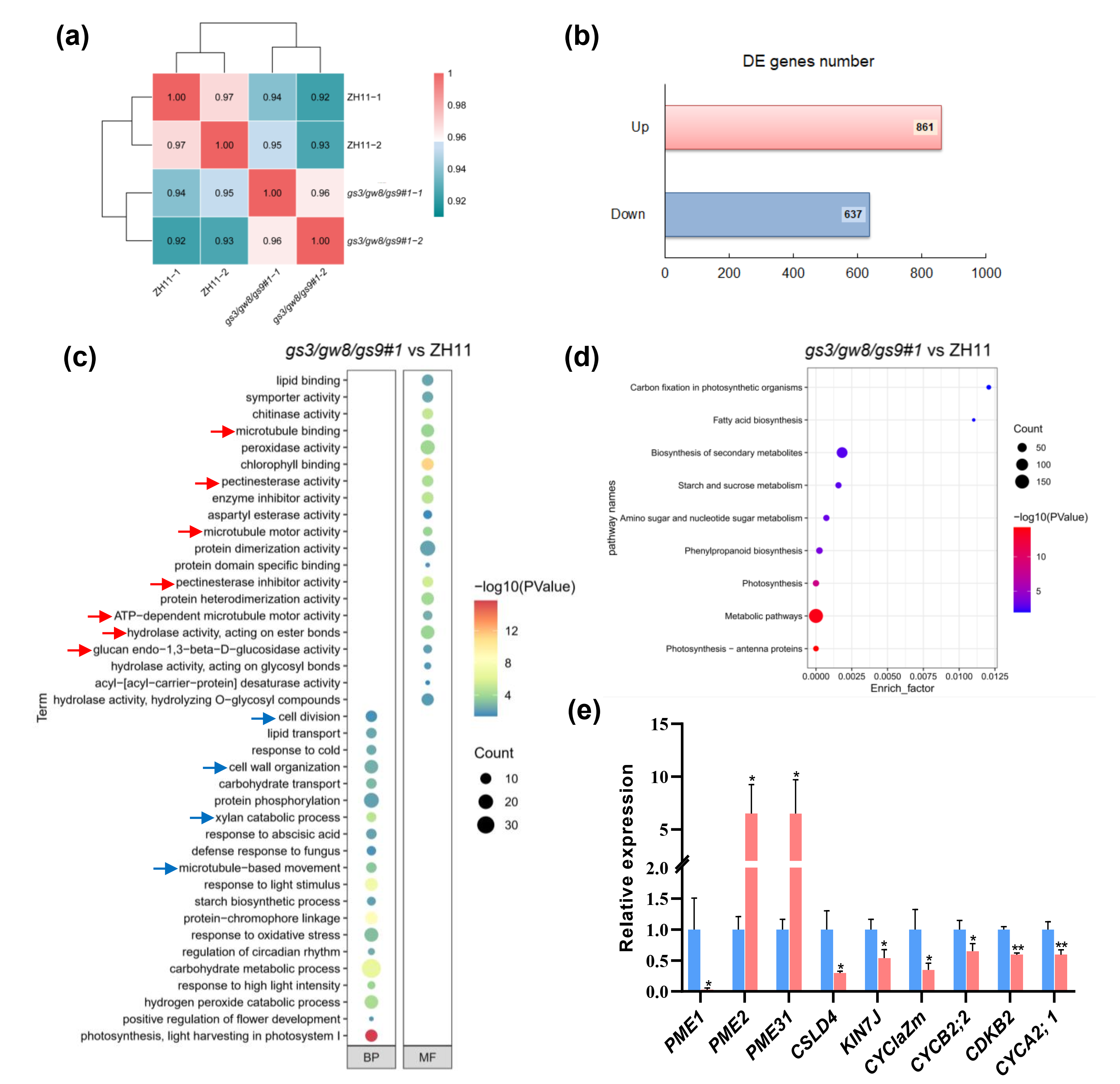


**Supplemental Figure 7. Transcriptome analysis of the pistil of ZH11 and the *gs3/gw8/gs9* mutant at stage 11.** (a) Pearson’s correlation coefficients for the two biological replicates. (b) Number of DEGs between the pistil of ZH11 and the gs3/gw8/gs9 mutant at stage 11. (c) Gene ontology (GO) classification of DEGs. *P* values were calculated by Fisher`s exact test. (d) KEGG pathway of differential expression genes (DGEs). (e) RT-qPCR analyses of nine selected DEGs involved in cell division and cell wall organization. “*” and “**” indicate statistical significance at *p* < 0.05 and *p* < 0.01 respectively. Data are shown as means ± S.E.M (n = 3).


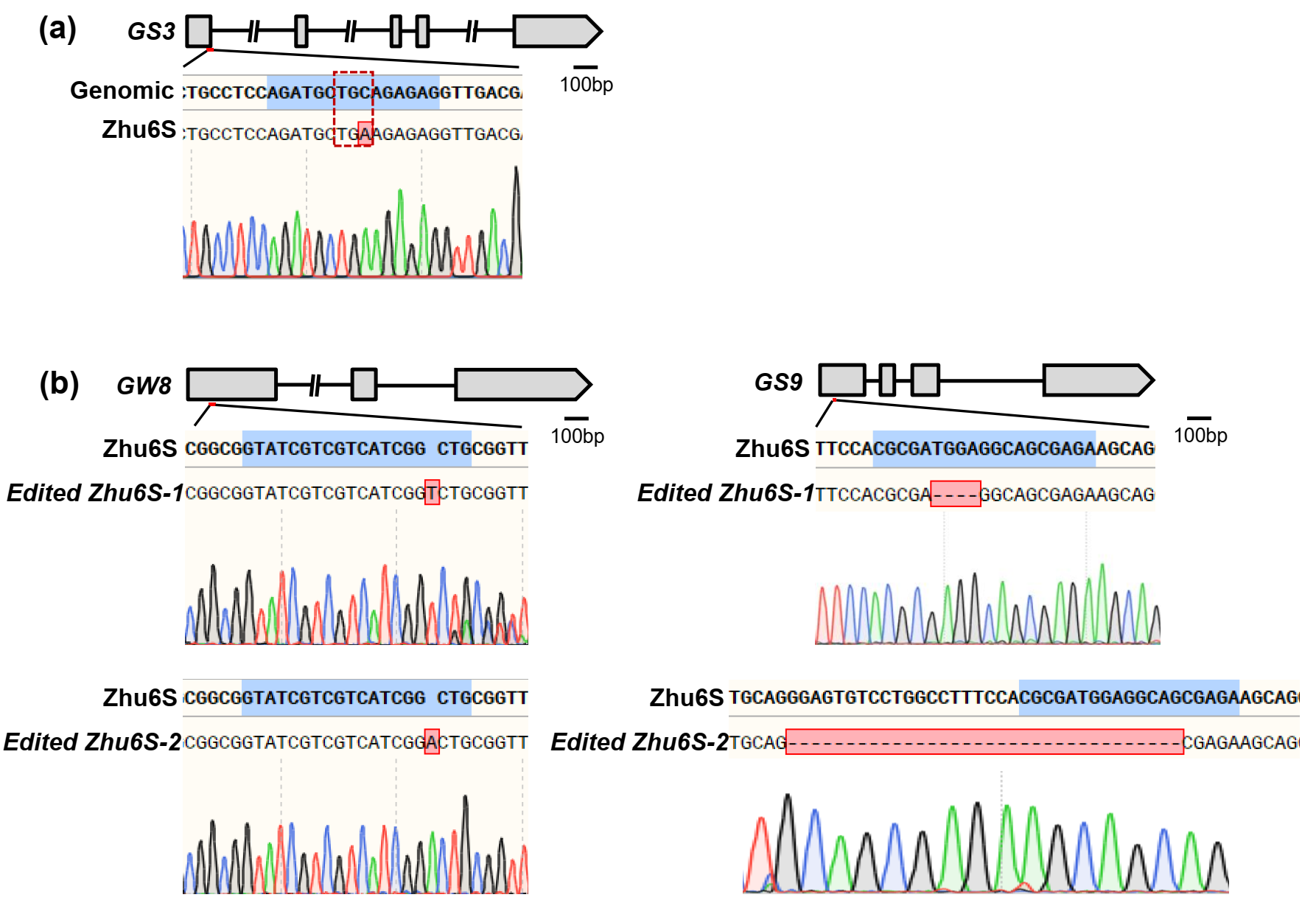


**Supplemental Figure 8. *GW8* and *GS9* mutation in Zhu6S generated by the CRISPR/Cas9 technology.** (a) No function *GS3* allele in Zhu6S. (b) Sequence analysis of knock-out mutants for *GW8 and GS9* in the Zhu6S background*.* The target sites are shown with light-blue color, and the mutated information is highlighted with pink boxed.


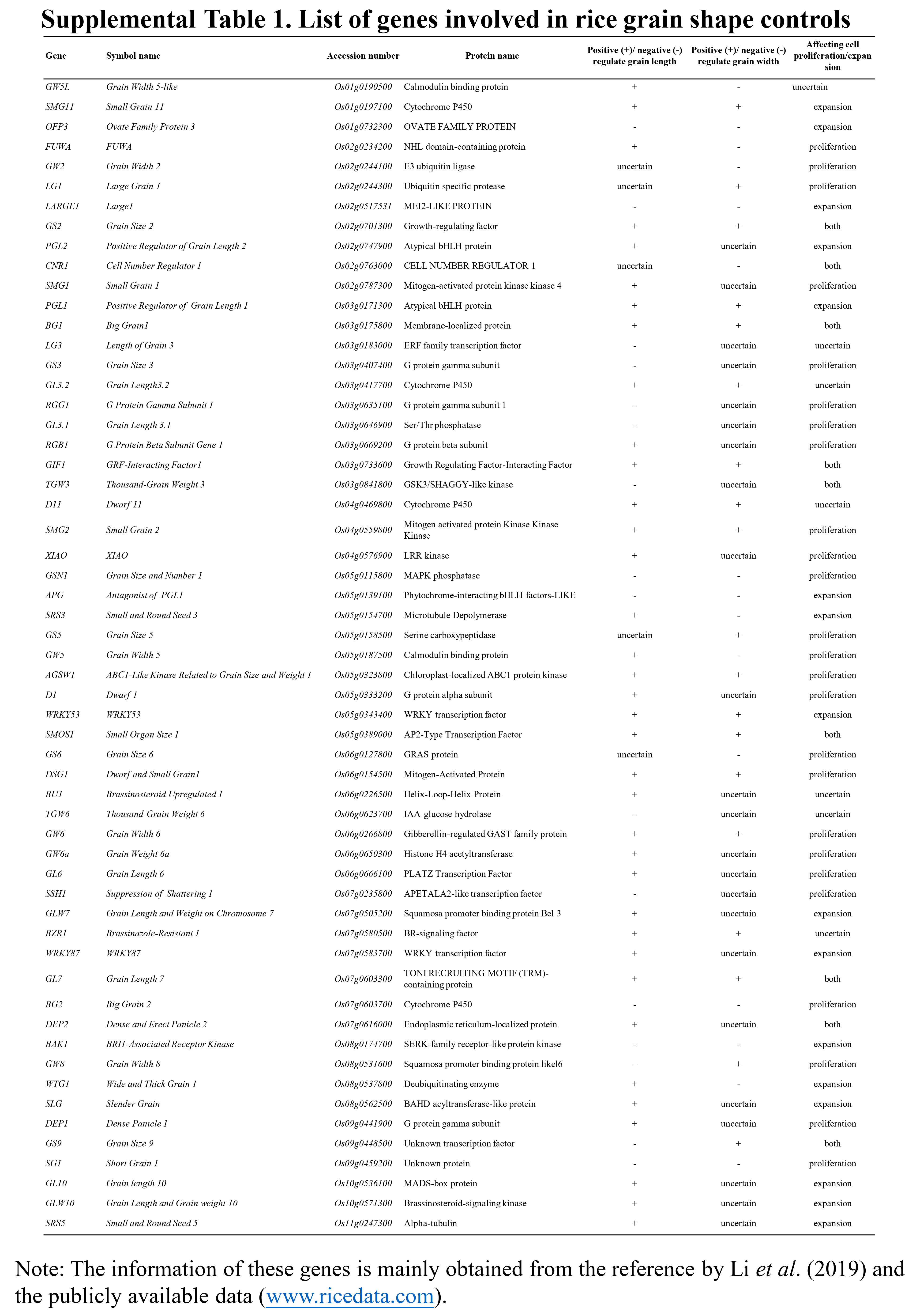


**
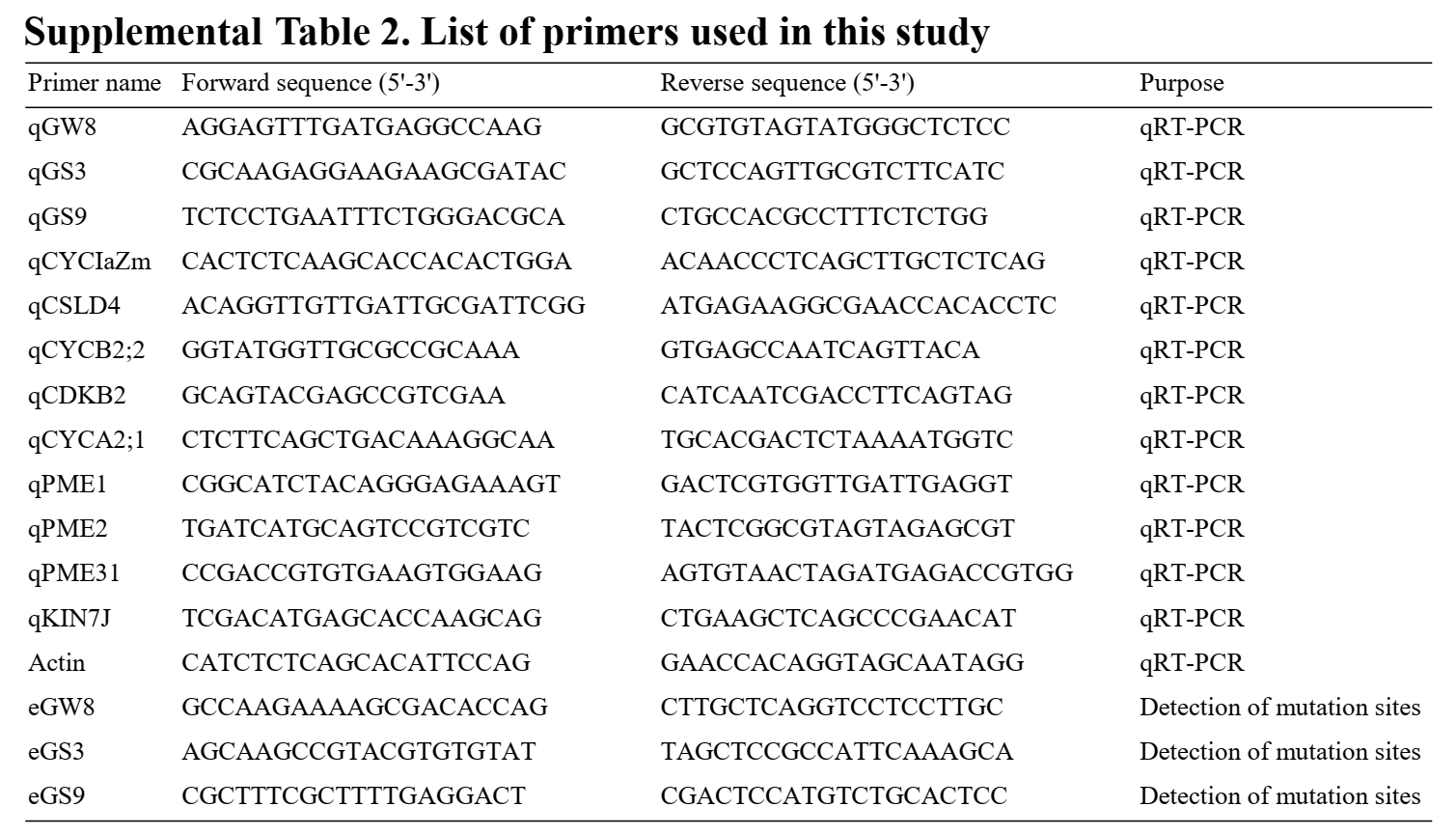
**
